# Local Application of Acibenzolar-*S*-Methyl Treatment Induces Antiviral Responses in Distal Leaves of *Arabidopsis thaliana*

**DOI:** 10.3390/ijms25031808

**Published:** 2024-02-02

**Authors:** Seiya Ito, Kagari Sakugawa, Fawzia Novianti, Tsutomu Arie, Ken Komatsu

**Affiliations:** 1 Graduate School of Agriculture, Tokyo University of Agriculture and Technology (TUAT), Fuchu 183-8509, Japanarie@cc.tuat.ac.jp (T.A.); 2 Institute of Global Innovation Research (GIR), Tokyo University of Agriculture and Technology (TUAT), Fuchu 183-8509, Japan

**Keywords:** systemic acquired resistance, plant virus, acibenzolar-*S*-methyl, plant activator, salicylic acid, *N*-hydroxypipecolic acid

## Abstract

Systemic acquired resistance (SAR) is a plant defense mechanism that provides protection against a broad spectrum of pathogens in distal tissues. Recent studies have revealed a concerted function of salicylic acid (SA) and *N*-hydroxypipecolic acid (NHP) in the establishment of SAR against bacterial pathogens, but it remains unknown whether NHP is also involved in SAR against viruses. We found that the local application of acibenzolar-*S*-methyl (ASM), a synthetic analog of SA, suppressed plantago asiatica mosaic virus (PlAMV) infection in the distal leaves of *Arabidopsis thaliana*. This suppression of infection in untreated distal leaves was observed at 1 day, but not at 3 days, after application. ASM application significantly increased the expression of SAR-related genes, including *PR1*, *SID2*, and *ALD1* after 1 day of application. Viral suppression in distal leaves after local ASM application was not observed in the *sid2-2* mutant, which is defective in *isochorismate synthase 1* (ICS1), which is involved in salicylic acid synthesis; or in the *fmo1* mutant, which is defective in the synthesis of NHP; or in the SA receptor *npr1-1* mutant. Finally, we found that the local application of NHP suppressed PlAMV infection in the distal leaves. These results indicate that the local application of ASM induces antiviral SAR against PlAMV through a mechanism involving NHP.

## 1. Introduction

Plants have evolved a sophisticated immune system to defend themselves from microbes. Effective plant defense involves dynamic responses triggered by the recognition of microbial molecular patterns and secreted proteins, known as pattern-triggered immunity (PTI) and effector-triggered immunity (ETI), respectively [1]. The local activation of PTI or ETI primes immunity in the distal tissues. This phenomenon, called systemic acquired resistance (SAR), protects plants from further infection by a broad spectrum of pathogens [2]. Establishment of SAR by local activation of PTI or ETI requires long-distance signaling between local and distal plant tissues [3].

SAR is associated with transcriptional changes in distal leaves that are dependent on two endogenous chemicals, salicylic acid (SA) and *N*-hydroxypipecolic acid (NHP) [4]. SA, biosynthesized by the isochorismate synthase (ICS) and phenylalanine ammonia lyase pathways, is a well-recognized key regulator of plant immunity to biotrophic pathogens. Isochorismate synthase 1 (ICS1) in the ICS pathway is induced by microbe recognition and is primarily responsible for SA production in the model plant Arabidopsis (*Arabidopsis thaliana*) [5]. While SA accumulation alone is insufficient to explain SAR establishment, NHP, produced from its precursor pipecolic acid (Pip) by flavin-dependent monooxygenase 1 (FMO1), is essential for SAR as a mobile signal [6,7]. Loss of Pip accumulation caused by the disruption of the *AGD2-like defense response protein 1* (*ALD1*) gene results in SAR deficiency, supporting the importance of NHP in SAR activation [7,8]. Recent studies have elucidated the concerted function of SA and NHP in SAR [9,10]. Both the NHP and SA biosynthetic pathways, including the *FMO1* and *ALD1* genes, are inducible by pathogen infection, and this induction requires the SA receptor nonexpressor of pathogenesis-related genes1 (NPR1) [11].

In addition to pathogen-induced SAR, the exogenous treatment of plants with SA or NHP can activate SAR, resulting in diminished pathogen growth in distal leaves [9,11]. The induction of SAR by NHP treatment is significantly reduced in the ICS1-defective *sid2* and *npr1* mutants, corroborating the importance of the positive crosstalk between NHP and SA [11], although NHP treatment also activates SAR in a partially SA-independent manner [11,12]. However, as recent findings regarding the NHP’s critical role in SAR establishment have been derived from studies using a few pathogen species, such as the bacterial pathogen *Pseudomonas syringae* and the oomycete pathogen *Hyaloperonospora arabidopsidis*, it remains uncertain whether NHP also plays an important role in SAR against other types of plant pathogens, including viruses, in Arabidopsis.

SAR can also be induced via treatment with an artificial compound, acibenzolar-*S*-methyl (ASM), also called benzothiadiazole *S*-methyl ester (BTH), a synthetic analog of SA [13]. ASM is well known as a plant immune activator, inducing defense responses against various pathogens, including fungi, bacteria, and viruses, without directly affecting microbes and viruses [14]. As the most studied plant activator, ASM has been tested in numerous pathosystems and shown to protect against a wide variety of diseases under laboratory conditions; it has also been successfully used in the field to control fungal and bacterial diseases in crops [15]. However, most studies have applied ASM or other plant activators to whole plants, which has not allowed researchers to clarify whether their effects on pathogens are local or systemic. To the best of our knowledge, no study has rigorously investigated SAR against plant viruses in Arabidopsis as a systemic effect by distinguishing between plant-activator-treated and virus-inoculated leaves.

We recently demonstrated that ASM limits the extent of infection of an RNA virus, plantago asiatica mosaic virus (PlAMV), in Arabidopsis in an NPR1-dependent manner [16,17]. However, information on the mechanism of action of plant immune activators on viruses remains vague, such as whether the effect of plant immune activators on viruses is limited to the treated tissues or if they can also induce antiviral responses in distal tissues. In this study, we found that the local application of ASM to specific leaves of Arabidopsis induces SAR in untreated leaves and suppresses virus infection. Additionally, virus inoculation assays in Arabidopsis mutants implicated ICS1, FMO1, and NPR1 in ASM-induced SAR against viruses. Finally, we found that local NHP treatment significantly reduces virus accumulation in the distal leaves in wild-type Arabidopsis. These findings indicate that antiviral responses induced in the SAR are activated by ASM and NHP and that the signaling pathway that leads to this antiviral SAR, which involves NPR1, is comparable to the pathway involved in the well-studied antibacterial responses.

## 2. Results

### 2.1. ASM Treatment of a Single Leaf Suppresses PlAMV Infection

**Figure 1:**
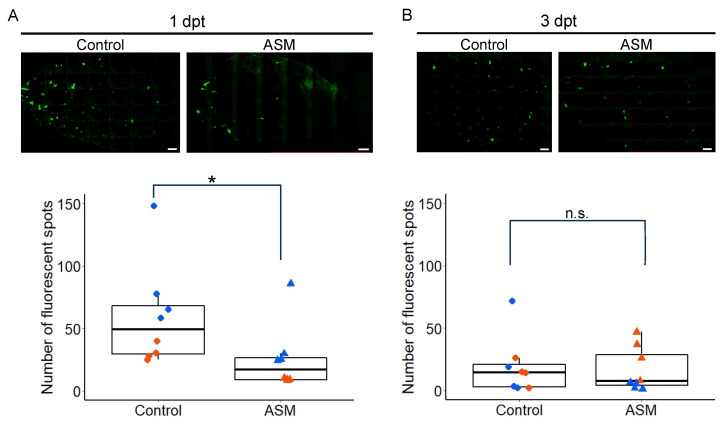
Acibenzolar-*S*-methyl (ASM) treatment of one leaf transiently suppresses green fluorescent protein (GFP)-expressing plantago asiatica mosaic virus (PlAMV-GFP) infection on the treated leaf. PlAMV-GFP was inoculated at one day (**A**) or three days (**B**) after ASM or water treatment. GFP fluorescence of PlAMV-GFP-inoculated leaf of wild-type *Arabidopsis thaliana* at 2 days after inoculation, pretreated with ASM or water (control), is shown in upper panels. Scale bars = 1 mm. The number of fluorescent spots on each leaf at 2 days after inoculation of PlAMV-GFP on 1 mM ASM-treated or control leaves is shown in lower graphs. Data consist of two independent experiments, each with four leaves represented using different colors. Asterisk indicates a significant difference between ASM-treated and untreated control leaves (* *p* < 0.05; n.s., not significant).

A previous study showed that spray treatment of all the rosette leaves of Arabidopsis Col-0 plants with 1 mM ASM suppressed PlAMV infection in the treated leaves at 3 days post-treatment (3 dpt) [17]. To investigate whether PlAMV infection could also be suppressed by ASM treatment of a single leaf, we pretreated the sixth leaf of four-week-old Col-0 plants with 1 mM ASM or water (control) and inoculated the same leaf with virions of green fluorescent protein (GFP)-expressing PlAMV (PlAMV-GFP) at 1 or 3 dpt. The number of fluorescent spots formed in the leaves was measured at two days after inoculation. Similar to the treatment of all the rosette leaves, ASM treatment of a single leaf significantly reduced the number of fluorescent spots in the leaf inoculated with PlAMV-GFP at 1 dpt (Figure 1A). However, when PlAMV-GFP was inoculated at 3 dpt with ASM, there was no significant reduction in the number of fluorescent spots compared with water treatment (Figure 1B). This result indicates that the suppression of PlAMV-GFP infection by ASM occurs even when ASM is applied to a single leaf but that the suppression is only effective at 1 dpt.

### 2.2. Local Treatment with ASM Suppresses PlAMV Infection in Untreated Distal Leaves

The above result, combined with the results of our previous study [17], suggests that the duration of suppression of PlAMV-GFP infection was prolonged when multiple leaves were treated with ASM. This led us to hypothesize that there is a synergistic antiviral response in each leaf of the plant whose rosette leaves were all treated with ASM, which could be mediated by an induction of antiviral responses in distal and untreated leaves.

**Figure 2:**
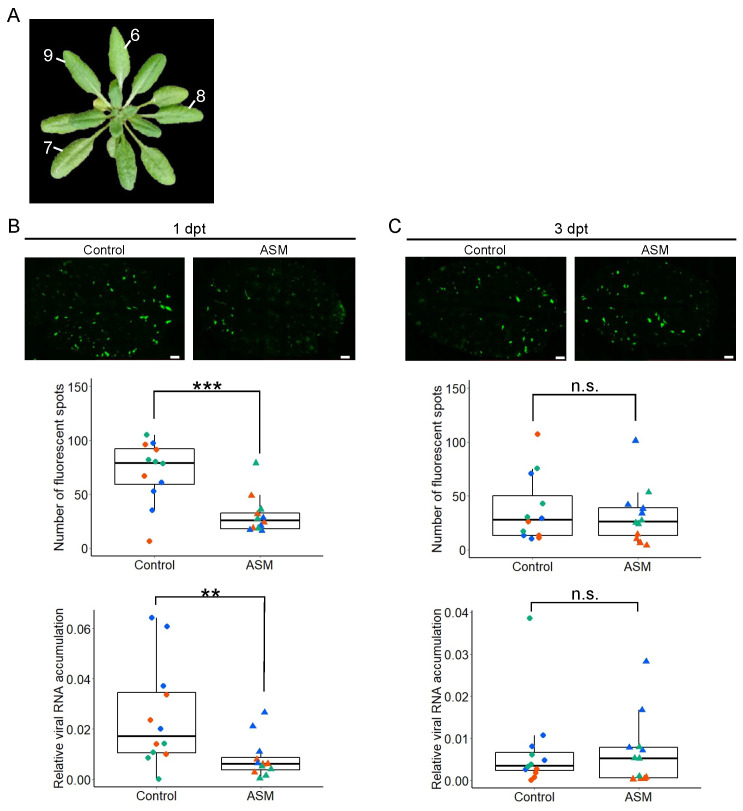
Local treatment with ASM transiently suppresses PlAMV-GFP infection in untreated systemic leaves. (**A**) Experiments with the systemic leaves of Arabidopsis plants with local pretreatment with ASM. The local pretreatment with ASM or water (control) was performed on the 6th, 7th, and 8th leaves, and the 9th leaf was inoculated with PlAMV-GFP at 1 or 3 days post-treatment (dpt). (**B**) The effect of local pretreatment with ASM or water (control) at one day before virus inoculation on PlAMV-GFP infection. GFP fluorescence of PlAMV-GFP-inoculated leaf (top panel), the number of fluorescent spots on each leaf (middle graph), and the relative viral RNA accumulation analyzed using reverse-transcription quantitative PCR (RT-qPCR) with *AtActin2* as a reference gene (bottom graph) of wild-type Arabidopsis at 2 days after inoculation. Scale bars in the top panels = 1 mm. (**C**) The effect of local pretreatment with ASM or water (control) at three days before virus inoculation on PlAMV-GFP infection. Data consist of three independent experiments, each with four leaves represented using different colors. Asterisk indicates a significant difference between ASM-treated and untreated control leaves (*** *p* < 0.001 and ** *p* < 0.01; n.s., not significant). Scale bars in the top panels = 1 mm.

To determine whether ASM treatment also suppressed virus infection in untreated leaves, the sixth, seventh, and eighth leaves of Col-0 were treated with ASM or water, and the upper ninth leaf was inoculated with PlAMV-GFP 1 or 3 dpt (Figure 2A). Two days after inoculation, GFP fluorescence in the inoculated leaves was observed, and the number of fluorescent spots was measured (Figure 2B,C). When PlAMV-GFP was inoculated into the upper leaf at 1 dpt with ASM for the three lower leaves, the number of fluorescent spots significantly reduced (Figure 2B). On the other hand, when PlAMV-GFP was inoculated at 3 dpt with ASM, there was no significant difference in the number of fluorescent spots between ASM-treated and water-treated plants (Figure 2C).

To examine viral RNA accumulation under the same conditions, leaves were harvested 2 days after inoculation, and viral RNA accumulation was measured using reverse-transcription quantitative PCR (RT-qPCR). The results showed that pretreatment of the lower leaves with ASM 1 day prior to PlAMV-GFP inoculation significantly reduced viral RNA accumulation in the distal untreated leaf compared to the control treatment. However, pretreatment with ASM 3 days prior to inoculation had no effect on virus accumulation (lower graphs in Figure 2B,C), which is consistent with the results obtained by counting the number of fluorescent spots. These results indicate that local treatment with ASM can suppress PlAMV-GFP infection on distal leaves and that this effect is transient and lasts only one day.

### 2.3. Local Treatment with ASM Upregulates SAR-Related Genes in Untreated Distal Leaves

**Figure 3:**
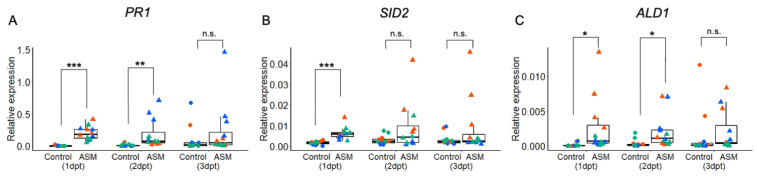
SAR-related genes are upregulated in a distal systemic leaf after ASM treatment of the lower leaves. Expressions of *PR1* (**A**), *SID2* (**B**), and *ALD1* (**C**) genes in the 9th leaf of Arabidopsis Col-0 plants at 1, 2, and 3 dpt with water (control) or 1 mM ASM to the lower leaves (6th, 7th, and 8th leaves). Relative expression levels of each gene analyzed via RT-qPCR with *AtActin2* as a reference gene are shown. Data consist of three independent experiments, each with four leaves represented using different colors. Asterisk indicates a significant difference between ASM-treated and untreated control leaves (*** *p* < 0.001, ** *p* < 0.01, and * *p* < 0.05; n.s., not significant).

Our data suggest that local ASM treatment induces antiviral systemic acquired resistance (SAR), which may be associated with pathways similar to those previously reported to be induced in SAR by *P*. *syringae* in Arabidopsis. To determine whether ASM treatment upregulates the genes reported to be induced in SAR, we analyzed the expression levels of three SAR-related genes, *PR1*, *SID2*, and *ALD1,* in the distal leaves of plants pretreated locally with ASM at 1, 2, and 3 dpt. These three genes have been reported to be strongly induced during SAR [9,11]. The results showed that the expressions of *PR1*, *SID2*, and *ALD1* were significantly increased by ASM at 1 dpt compared to water treatment (Figure 3A–C). The expressions of *PR1* and *ALD1* was also significantly increased in ASM-pretreated plants at 2 dpt (Figure 3A,C). In addition, the expressions of *PR1*, *SID2*, and *ALD1* tended to be higher in the ASM-pretreated plants than in the control plants at 3 dpt. These results reveal that antiviral SAR induced by ASM is associated with the increased expression of SAR-related genes as reported in the previous studies employing bacterial pathogens.

### 2.4. ASM-Mediated Suppression of PlAMV Infection in Untreated Distal Leaves Depends on Biosynthesis of SA and NHP as Well as NPR1

**Figure 4:**
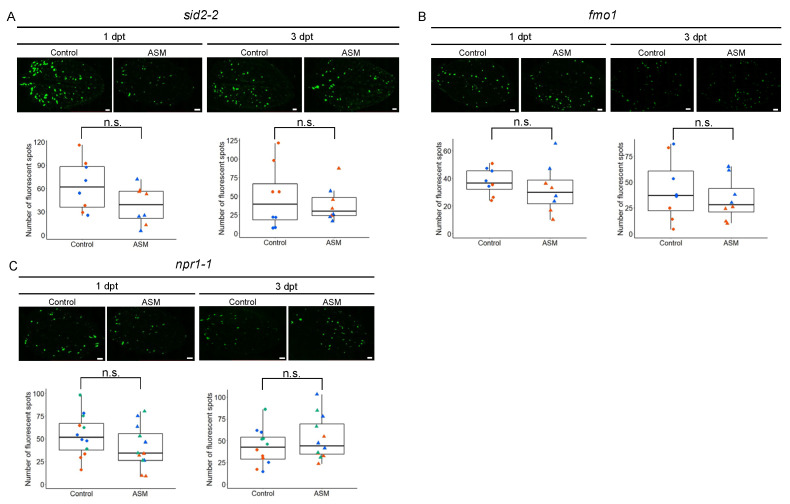
ASM-induced antiviral responses in upper distal leaves depend on biosynthesis of SA, NHP, and NPR1. Local pretreatment with ASM or water (control) was performed on 6th, 7th, and 8th leaves of Arabidopsis mutants *sid2-2* (**A**), *fmo1* (**B**), and *npr1-1* (**C**) at 1 or 3 days before inoculation of PlAMV-GFP to the 9th leaf. GFP fluorescence of PlAMV-GFP-inoculated leaf (upper panel), the number of fluorescent spots on each leaf (lower graph). Scale bars in the upper panels = 1 mm. Data consist of two (**A**,**B**) or three (**C**) independent experiments, each with four leaves represented using different colors. n.s., not significant by a statistical analysis.

In SAR, SA and NHP accumulate in uninfected tissues and induce defense responses against pathogens [7,13]. Consistent with this, the biological induction of SAR via inoculation with the avirulent bacterium *P. syringae* pv. *tomato* (Pst) in Arabidopsis requires the NHP biosynthesis gene *FMO1* [18]. A recent study using the Pst and Arabidopsis pathosystem showed that NHP-triggered SAR partially requires ICS1, which is involved in the synthesis of SA [11]. To determine whether SA and NHP biosynthesis is required for antiviral SAR in the suppression of PlAMV-GFP infection in untreated distal leaves, we tested the Arabidopsis *sid2-2* mutant, which lacks ICS1 function, and the NHP biosynthesis mutant *fmo1*. The results showed that there was no significant difference in the number of fluorescent spots of PlAMV-GFP between ASM-pretreated plants and control plants in either mutant at either 1 or 3 dpt (Figure 4A,B). Taken together, these results indicate that the ASM-mediated restriction of PlAMV infection in untreated distal leaves requires the biosynthetic pathway of SA and NHP.

In Arabidopsis, the biological induction of SAR via Pst inoculation and chemical induction of SAR by NHP treatment require the SA receptor NPR1 [11]. The suppression of PlAMV infection via ASM treatment of all the rosette leaves also requires NPR1 [17]. To determine whether NPR1 is involved in antiviral SAR against PlAMV, we tested the Arabidopsis *npr1-1* mutant. We found no significant difference in the number of GFP fluorescent spots between the ASM and water treatments at either 1 or 3 dpt (Figure 4C). This result confirms the function of NPR1 in suppressing PlAMV-GFP infection in untreated distal leaves through local ASM treatment.

### 2.5. Local Treatment with NHP Suppresses PlAMV Infection in Untreated Distal Leaves

**Figure 5:**
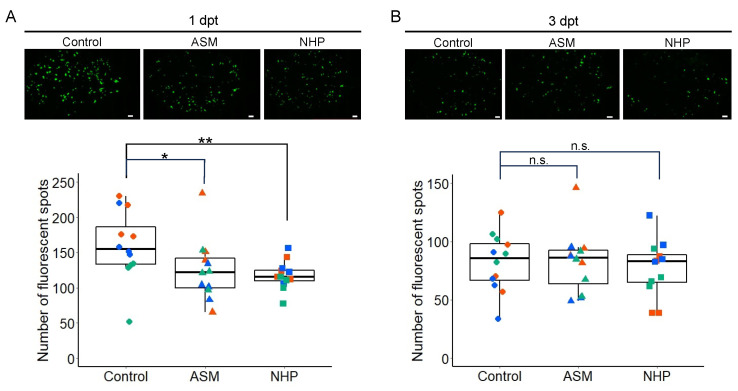
Local treatment with NHP transiently suppressed PlAMV-GFP infection in untreated systemic leaves. The local pretreatment with NHP, ASM (control), or water (control) was performed on 6th, 7th, and 8th leaves, and the 9th leaf was inoculated with PlAMV-GFP at 1 (**A**) or 3 days (**B**) after the treatment. GFP fluorescence of PlAMV-GFP-inoculated leaf (upper panel) and the number of fluorescent spots of each leaf (lower graph). Scale bars in the upper panels = 1 mm. Data consist of three independent experiments, each with four leaves represented using different colors. Asterisk indicates a significant difference between NHP- or ASM-treated and water-treated control leaves (** *p* < 0.01 and * *p* < 0.05; n.s., not significant).

The above results prompted us to examine whether local treatment with NHP could induce antiviral responses in distal untreated leaves, as it induces SAR against bacterial pathogen Pst, as reported previously [6,7]. We treated the sixth, seventh, and eighth leaves of Arabidopsis Col-0 plants with 1 mM NHP or water and inoculated the ninth leaves with PlAMV-GFP at 1 or 3 dpt. ASM was also used as a control in each experiment. At 2 days after inoculation of PlAMV-GFP, the number of fluorescent spots in the upper untreated leaves of plants pretreated with NHP and ASM was significantly reduced compared to that of the water control when PlAMV-GFP was inoculated at 1 dpt with NHP and ASM (Figure 5A). On the other hand, there was no significant difference in the number of fluorescent spots of PlAMV-GFP between NHP- and control-treated plants when PlAMV-GFP was inoculated at 3 dpt (Figure 5B). These results indicate that the NHP treatment of local leaves induces antiviral SAR, but this effect lasts only for one day, the same duration as the antiviral SAR induced via local ASM treatment.

## 3. Discussion

In this study, we revealed that antiviral SAR is induced in distant leaves via the local application of ASM, and the induction of this antiviral SAR requires the biosynthesis of both SA and NHP. Furthermore, we demonstrated that NHP alone is capable of triggering SAR against plant viruses in distant leaves. 

While SAR has historically been considered broad-spectrum [19], its initial discovery was reported in virus research [20]. However, recent molecular and genetic studies on SAR have predominantly focused on bacteria, with limited attention given to viruses. Nevertheless, pioneering work on plant immune activators, including probenazole (PBZ) and benzisothiazole (BIT), that induce SAR and exhibit antiviral effects in tobacco plants aligns well with the results of this study employing ASM [21]. But, it remains unclear whether plant-activator-mediated SAR in distant leaves can restrict viral infection in Arabidopsis and whether the involvement of NHP in SAR, as elucidated in the Arabidopsis–Pst system, is applicable to viruses.

To address this knowledge gap, we employed PlAMV, a positive-strand RNA virus that efficiently infects Arabidopsis, and ASM, a plant immune activator that suppresses this infection. Our results revealed that the expression of SAR-related genes, such as NHP and SA biosynthetic genes, was induced in distant leaves at 1 dpt with ASM, supporting the dependence of antiviral SAR on SA and NHP (Figure 3). Furthermore, our results demonstrate that ASM-activated antiviral SAR is dependent on *SID2*, *FMO1*, and *NPR1* (Figure 4). Our finding that the expression of *SID2* is necessary for ASM-activated antiviral SAR seems to contradict that of a prior study suggesting that ASM activates the SAR signaling pathway downstream of SA accumulation [22]. This can be explained by the requirement for endogenous SA in ASM-mediated antiviral responses in distal leaves but not in local, ASM-applied leaves. Indeed, endogenous SA biosynthesis in distal leaves is induced by NHP, which is required for the full establishment of NHP-triggered SAR [11]. Efficient inhibition of virus infection via ASM-induced SAR may need signal amplification through elevated SA accumulation by ICS1 in distal leaves, as observed previously in the case of Pip- and NHP-induced antibacterial SAR [7,11,12]. The molecular mechanisms by which ASM amplifies SAR signaling require further investigation.

Significantly, our study demonstrated for the first time that the local application of NHP resulted in a reduction in viral accumulation in the distal leaves. Similar to the effects of ASM [23], NHP induces SAR-associated transcriptional reprogramming, which requires the function of NPR1 [11]. These findings underscore the shared underlying mechanisms between antiviral and antibacterial SAR, both of which can be activated by NHP. Additionally, the application of NHP reduced the infection of the oomycete pathogen *H*. *arabidopsidis* in the distal leaves of Arabidopsis [7]. Collectively, plants may simultaneously activate multiple responses to combat three different types of pathogens, fungi, bacteria, and viruses, through pathways involving NHP. 

However, it is unlikely that the same NPR1 downstream pathway exerts identical inhibitory effects against all pathogen types, considering the evidence that the expression of the downstream genes of NPR1 in plant immunity diverges into multiple subsets [24]. For example, while a peptide derived from the well-known SA marker gene *PR1*, whose expression is strictly dependent on NPR1, is critical for SAR against bacteria in Arabidopsis, its effect on viruses has not been investigated [25]. Proteins that act in the apoplast, such as PR1, would have limited efficacy against viruses due to the strict confinement of plant viral infections within cells. Overall, only the specific subsets of host factors that affect intracellular processes may be effective against viruses in the context of SAR. 

SAR has traditionally been thought to be long-lasting, with effects persisting for several weeks [19,26]. Early reports indicated efficacy against viruses for at least 20 days [20], and recent studies on the Japanese radish (*Raphanus sativus* var. *longipinnatus*) and bacteria (*Pseudomonas cannabina* pv. *alisalensis*) indicated a sustained effect for at least one week [27]. However, in our study, the antiviral inhibitory effect induced by ASM or NHP was observed in the distal leaves after one day but not after three days, indicating a transient effect. This may be consistent with the findings of other studies of NHP in Arabidopsis, which commonly indicate an effect within one day. However, there are trends of lower numbers of fluorescent spots at 3 dpt than at 1 dpt in control leaves, which seems to mask the antiviral effect of ASM and NHP. Although we cannot explain the mechanism underlying this phenomenon, it is possible that age-related resistance is involved [28].

It is conceivable to prolong the duration of antiviral SAR effects through methods such as root application or treating a higher number of leaves, as employed in our prior ASM application study [17]. Indeed, the inhibitory effect against tobacco mosaic virus in systemic leaves persisted for up to 15 days after ASM treatment of whole tobacco plants, albeit with variations depending on the cultivar [29]. Further studies are needed to investigate whether the antiviral SAR in Arabidopsis is effective against other plant viruses and, if so, whether the effect is transient or not.

In conclusion, our study expands the scope of future research on SAR driven by NHP, encompassing viruses as significant targets for investigation. This emerging field of research holds the potential to contribute to unraveling how SAR effectively suppresses the propagation of pathogens with diverse lifestyles.

## 4. Materials and Methods

### 4.1. Plant Materials

Arabidopsis wild-type Col-0 plants, as well as its mutants *sid2-2* (CS16438), *fmo1* (SALK_02616163C), and *npr1-1* (CS3726), were grown in a mixture of vermiculite and perlite in a 4:1 ratio in a growth chamber under a 16/8 h light/dark cycle at 50–70% relative humidity and 22 °C after 3 days at 4 °C. Seeds of all mutant plants were provided by the Arabidopsis Biological Resource Center (ABRC; Columbus, OH, USA). 

### 4.2. Chemical Treatment

As described previously [17], acibenzolar-*S*-methyl (ASM) (50% (*w*/*w*) active ingredient; Syngenta, Tokyo, Japan) was dissolved in water containing 0.004% (*v*/*v*) Silwet L-77 (Nippon Unicar, Kawasaki, Japan) to a final concentration of 1 mM. Water containing 0.004% (*v*/*v*) Silwet L-77 was used as a control. In the PlAMV-GFP inoculation experiment on the ASM-treated leaves, 5 μL drops of 1 mM ASM were applied to the sixth leaf of Arabidopsis plants (Figure 2A). In the PlAMV-GFP inoculation experiment and SAR-related marker gene expression analyses in untreated systemic leaves after ASM treatment to the lower leaves, 5 μL drops of ASM were applied to the sixth, seventh, and eighth leaves of four-week-old Arabidopsis plants. NHP (MedChemExpress, Monmouth Junction, NJ, USA, HY-N7378) was dissolved in water containing 0.004% (*v*/*v*) Silwet L-77 to make a 1 mM solution, and 5 μL drops of the NHP solution were applied to a leaf.

### 4.3. Virus Inoculation and Fluorescence Observation

For virion preparation, PlAMV-GFP, a lily isolate of PlAMV expressing a green fluorescent protein (GFP) gene, was used to infect *Nicotiana benthamiana* via agroinfiltration [30]. Virus particles were purified from the infected plants showing mosaic symptoms, as described previously, and used for mechanical inoculation of *A. thaliana*. In the PlAMV-GFP inoculation experiment on untreated upper leaves, inoculations were made on the 9th leaf (one leaf above the three ASM-treated leaves) 1 or 3 days after ASM treatment (Figure 2A). 4 μL of PlAMV-GFP purified solution diluted to the concentration described previously [17] was inoculated. Two days after inoculation, each inoculated leaf was photographed using an HS all-in-one fluorescence microscope BZ-9000 (KEYENCE, Osaka, Japan), and the number of GFP fluorescent spots was measured manually.

### 4.4. RNA Extraction and RT-qPCR

One leaf was homogenized in liquid nitrogen, and total RNA was isolated using ISOGEN reagent (Nippon Gene, Tokyo, Japan) according to the manufacturer’s instructions. Each RNA sample was treated with RQ1 RNase-free DNase (Promega, Madison, WI, USA), and about 500 ng of the DNase-treated total RNA was reverse-transcribed using ReverTra Ace (TOYOBO, Tokyo, Japan). cDNA corresponding to 20 ng of total RNA was used for quantitative PCR (qPCR) using a Thermal Cycler Dice Real Time System II MRX [TP960] (Takara-Bio, Kusatsu, Japan) with Go Taq qPCR Master Mix (Promega, USA). *AtActin2* was used as a reference. The primer sets used for RT-qPCR were as follows: AtActin2-F (5′-AGTGTCTGGATCGGTGGTTC-3′) and AtActin-R (5′-CCCCAGCTTTTTAAGCCTTT-3′) for *AtActin2* [31]; AtPR1-F (5′-CGGAGCTACGCAGAACAACT-3′) and AtPR1-R (5′-CTCGCTAACCCACATGTTCA-3′) for *PR-1* [32]; AtSID2-F (5′-TCCGTGACCTTGATCCTTTC-3′) and AtSID2-R (5′-ACAGCGATCTTGCCATTAGG-3′) for *SID2* [33]; AtALD1-F (5′-GTGCAAGATCCTACCTTCCCGGC-3′) and AtALD1-R (5′-CGGTCCTTGGGGTCATAGCCAGA-3′) for *ALD1* [34]; PlAMV-3877F (5′-CCTCATTCTCCCTGCTGAAG-3′) and PlAMV-4010R (5′-CTTGAGGGGGTCTTTGATGA-3′) for PlAMV genomic RNA [35]. 

### 4.5. Statistical Analysis

The data obtained by counting GFP fluorescence and RT-qPCR were fit to a mixed linear model using the lme4 package version 1.1-29 in the R environment version 4.0.3, as previously described [33]. Independent replicates of the experiment were set as random factors, and ASM or water treatment was set as a fixed factor. The mean estimates of the fixed factors were used for the *t*-test with the lmerTest package version 3.1-3.

## Data Availability

We will provide data supporting the reported findings in this article upon reasonable request.
